# Assessment of the Knowledge of Diabetes Mellitus Among School Teachers within the Scope of the Managing Diabetes at School Program

**DOI:** 10.4274/Jcrpe.756

**Published:** 2012-12-19

**Authors:** Zehra Aycan, Aşan Önder, Semra Çetinkaya, Hatice Bilgili, Nurdan Yıldırım, Veysel Nijat Baş, Havva Nur Peltek Kendirci, Sebahat Yılmaz Ağladıoğlu

**Affiliations:** 1 Dr. Sami Ulus Obstetrics and Gynecology Pediatric Health and Disease Training and Research Hospital, Pediatric Endocrinology Clinic, Ankara, Turkey

**Keywords:** diabetes mellitus, teacher education, schools

## Abstract

**Objective:** Training teachers and education professionals on diabetes is crucial for full-time monitoring of diabetic children in schools. The objective of this study was to assess the knowledge on diabetes in a group of school teachers in Turkey.

**Methods:** Between November 2010 and November 2011, 1054 teachers from three regions of Ankara were given a questionnaire to assess their knowledge on diabetes. The mean age of the group (27% males, 73% females) was 38.8±8 years. 61.7% of the participants were class teachers, 23.3% were school counselors, and the rest were physical education teachers and administrators.

**Results:** A fair percentage (47.6%) of the participants had a moderate knowledge level on diabetes and 32.4% expressed a lower level of knowledge. A large proportion (94%) gave an accurate definition of diabetes. Of the total group of 1054 teachers, 625 were aware that blood glucose level might decrease in diabetic children during follow-up. Also, 75% believed that diabetic children were eligible for physical education classes. 52.8% of these teachers had no diabetic child in their classes and teachers with a diabetic patient in their family had better knowledge of diabetes compared to their counterparts.

**Conclusions:** Our study results indicate that school teachers have limited knowledge on diabetes. We believe that their knowledge levels can be improved by widespread training programs.

**Conflict of interest:**None declared.

## INTRODUCTION

Type 1 diabetes mellitus (DM) is a major chronic disease with an incidence ranging from 64/100 000 to 0.1/100 000 per year in different countries (1). A study involving school-aged children in Istanbul, Turkey has shown a prevalence of0.67/1 000 (2). The prevalence of type 2 DM has also shown an increase in school-aged children and is reported to account for 10-40% of adolescent diabetics (3). DM is a condition which requires continuous monitoring of the patients. Early diagnosis and improved management will reduce the risk of complications of the disease. Given the fact that school-aged children spend the greater part of their time in school, the teachers can play a critical role in the monitoring of diabetic school children. At present in Turkey, responsibility for the management of diabetic children of school age is often undertaken by the children and their parents. Thus, there is a need to meet the demands of these children with a robust infrastructure in schools: a task, which can be realized by professional educators. To this end and in collaboration with the Turkish Pediatric Endocrinology and Diabetes Society, the Turkish Ministry of Health and the Turkish Ministry of National Education have designed a protocol which aims to increase awareness of diabetes in schools. The “Managing Diabetes at School Program” came into effect on April 2010 in Turkey. The main aim of this program was to increase the awareness of teachers on the needs of the diabetic child and thus to improve their support to these children in school. Education sessions on diabetes for teachers were held in several schools in Ankara. In this study, we aimed to assess the knowledge and attitudes of a group of school teachers towards diabetes before they were given any education about the disease. To our knowledge, no other study investigating the knowledge and attitudes of school teachers towards diabetes is available in Turkey.

## METHODS

In accordance with the Managing Diabetes at School Program, a training program was designed by education professionals. This program and detailed information on DM were also launched at www.okuldadiyabet.org, an official website. A total of 1500 teachers of varying socio-cultural status and working in primary and secondary schools in different regions of Ankara were given a questionnaire to assess their knowledge and attitudes towards diabetes. The mean age of the participants (27% males, 73% females) was 38.8±8 years. Forty-five percent had been working as a teacher for 11-15 years. Of the participants, 61.7% were class teachers, 23.3% school counselors, 7.5% physical education teachers, and 4.3% were administrators. The sociodemographic characteristics of the participants are shown in Table 1.

Subsequently, the teachers were included in a training program conducted by the pediatric endocrinologists working in our hospital. The program included several topics including DM definition, clinical signs and symptoms, emergency management of diabetes complications, and information on the attitudes of teachers, administrators and other peers. The responses of 1054 subjects were included in this presentation. 

## RESULTS

Of participants, 47.6% had a moderate level of knowledge on diabetes and 32.4% had less knowledge. A total of 125 subjects (11.8%) reported that they had a well-established knowledge on diabetes and 3.8% expressed a very well-established knowledge on the disease. Forty of the teachers (3.7%) had no knowledge about the disease. Four subjects did not answer the question. Regarding their sources of information, 35.5% reported that they had this information in the context of their general knowledge. Other sources included television in 7.9%, personal interest in 6.8%, contact with healthcare professionals in 4.2%, specific training in 1.3%, and other media in 7.6%. No source was given by 3.4% of the participants, while 28.3% did not answer the question. Diabetes was defined as an increased blood glucose level by 69% of participants, while 6.1% defined it as a decreased glucose level. The remaining subjects did not answer the question. Of participants, 94% accurately reported the symptoms of the disease as polydipsia, polyuria, fatigue, and dry mouth. Others mentioned joint pain, headache, skin rash, and ingrown nails. A total of 625 subjects (59.2%) were aware that blood glucose level might decrease, while 64 (6%) reported that no change would be observed in the glucose level during follow-ups. A total of 353 subjects (33.8%) did not answer the question. As to information on the onset age of the disease, 940 (89.1%) reported that it might develop at any age. A total of 798 (75.7%) participants noted that diabetic children could attend the physical education course. A total of 212 (20.1%) declared that they had no knowledge on this question, while 40 (3.7%) reported that diabetic children were not eligible to attend physical education classes. Four subjects (0.3%) did not answer the question.

In case of a decreased blood glucose level, 592 participants (56.1%) stated that they would give sugar-added products, while 212 (20.1%) would give a bar of chocolate or candy. Of participants, 17.9% did not know what to do in case of emergency, while the remaining subjects reported that they would give salt-added yoghurt or would slap the child’s face. With respect to the presence of diabetic children in the class and attitudes toward them (Table 2), 52.8% reported that no diabetic children were present in the class, while 23.5% did not answer the question. 89.9% declared that diabetic children might attend their class, while 0.4% stated that they would not give their consent to have diabetic children in their classes, and 63 (5.9%) noted that they would accept these children unwillingly, while 38 (3.6%) did not answer the question. In answer to the question of “What did you feel when you were first informed about diabetic children in your class?”, 788 subjects had no answer. Eighty seven (8.2%) reported that they felt sad, while 105 (9.9%) felt anxious. A total of 807 (76.5%) declared that they would give support to these children.

## DISCUSSION

It is well-known that micro- and macrovascular complications of DM can be prevented through a well-managed metabolic control (4). Good metabolic control can be only achieved with a comprehensive training. Schools are the major application areas of training in childhood diabetes and school professionals can play a critical role in the management of the disease. The ‘Managing Diabetes at School Program’ aims to increase the awareness of the teachers on diabetes. To increase the awareness of childhood diabetes, it is critical to expand the management strategies to every area, other than healthcare institutions, to which children attend.

In this study, almost all of the subjects defined the disease accurately, while they reported that they had a limited knowledge about the disease. This finding indicates a lack of information among the teachers regarding chronic diseases which may develop in every child in the class (5,6). Among teachers, 10.1% were unwilling to teach diabetic children, while 24.3% believed they should not allow the children to attend physical exercise classes, therefore, they ignored the beneficial effects of exercise on blood glucose regulation. This may be explained by limited information and anxiety for being responsible for these children. In other studies, it was reported that half of school professionals rejected using glucagon, while 47% felt its use unsafe for these children, an opinion consistent with the literature data (7,8). The major problems, as reported by the teachers, included emergency cases, non-attendance and legal issues (9,10). Increasing knowledge on the management of diabetes through training programs and eliminating anxiety regarding chronic diseases may remove the misunderstandings about diabetic children in schools. In addition, proper management of diabetes may increase the school attendance ratio and success rate of these children.

When information sources were considered, 35.5% of our subjects reported that they had acquired their information in the context of their general education. In another study involving teachers (11), school nurses were reported to be the major source of information for chronic diseases, since nurses also had a limited time to learn about the management of chronic diseases and were required to obtain disease-specific information as concisely as possible. In Turkey, only a few schools provide healthcare service. As a result, parents are usually responsible for their children’s health also during their school years. We therefore believe that training programs designed to address school teachers and administrators and conducted by specialized medical staff may be the most practical way to contribute to the care of these children. Gesteland et al (9) reported that group trainings were not efficient for the management of chronic diseases. In our study, we did not have any data on post-training assessments. We believe that large-scale studies are required to assess the long-term effect of teacher training on the management of chronic diseases. In addition, institution of continuous training programs in diabetes should also be considered (12).

The training and management of DM should target behavioral changes in the patients, in the parents and other caring individuals (12). A well-established relationship should be built among the patient, his/her parents and teachers. In a study, it was reported that teachers were incapable of making connections between a specific problem and the reasons underlying the problem, unless they were aware of the medical condition of the student (13). Westbom (14) reported that a higher number of children with chronic diseases were exposed to unfriendly behaviors at school with diminished communication with their peers. Also, it was shown that children with an impaired blood glucose regulation and non-compliance to the dietary recommendations were neglected by their teachers (15,16). In our study, the rate of teachers who were willing to give support to diabetic children was 76.5%. Therefore, we can conclude that an important ratio of the teachers will attend and benefit from training programs designed for them. However, studies are required to establish the success rate of these programs and their effects on the relationships at school.

Until today, several school programs worldwide, targeting management of DM, prevention of DM type 2, and lifestyle interventions were performed successfully (17,18). As the ‘Managing Diabetes at School Program’ is the first attempt in Turkey, the outcomes of this program should be analyzed carefully to develop new strategies based on different perspectives.

In conclusion, in this study, the responses to the questionnaire indicate that teachers in Turkish schools have limited information on diabetes and some worries on the management of the disease. By accessing large communities through teacher training programs, early diagnosis of childhood diabetes and also prevention/delaying of its complications may be achieved. 

## Figures and Tables

**Table 1:**
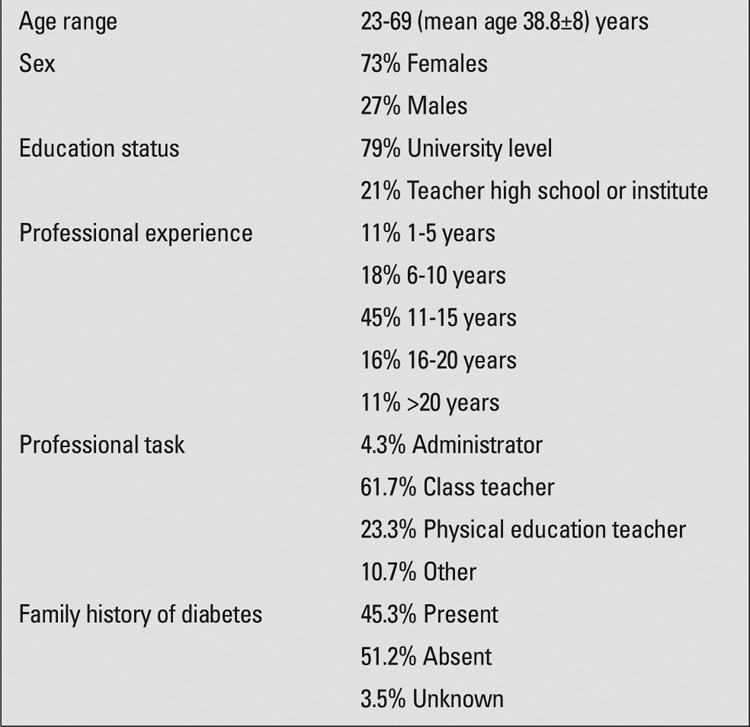
Sociodemographic characteristics of the subjects

**Table 2:**
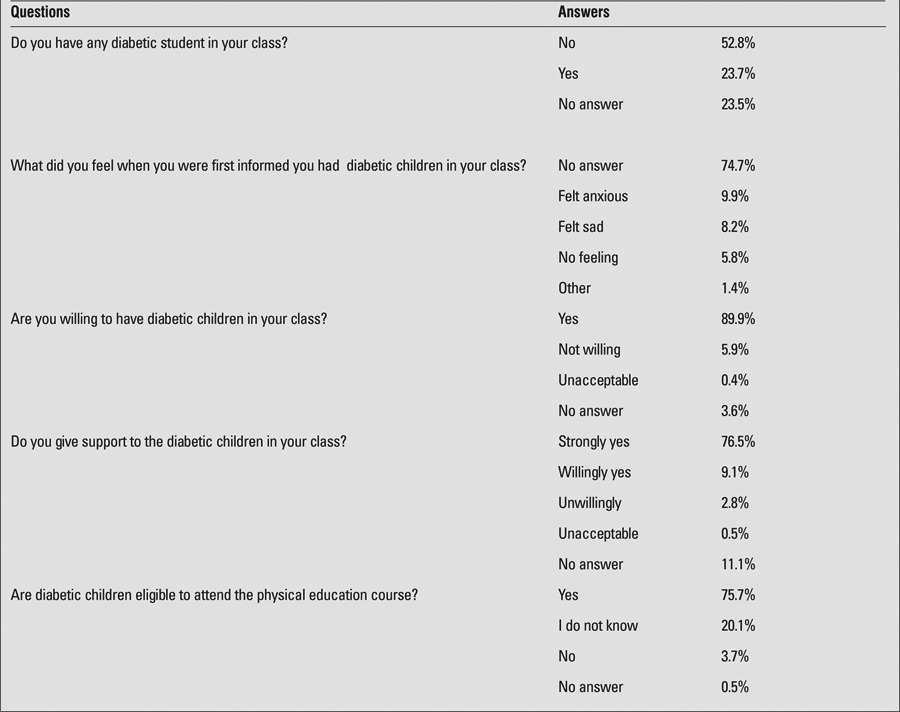
Questionnaire given prior to the training program
